# Is the Concept of Central Nervous System Immune Privilege Irrelevant in the Setting of Acute Infection?

**DOI:** 10.3389/fonc.2015.00099

**Published:** 2015-04-28

**Authors:** Amanda K. Huber, David N. Irani

**Affiliations:** ^1^Department of Neurology, University of Michigan Medical School, Ann Arbor, MI, USA

**Keywords:** central nervous system, infection, host immunity, viruses, bacteria

While historically viewed as an immune-privileged area fully isolated from the immune system, the central nervous system (CNS) is now appreciated to maintain dynamic bi-directional communication with the immune system across the blood–brain barrier (BBB) (1, 2). In no setting can this communication be more urgent that acute CNS infection – a dampened or delayed host response could allow an invading virus or bacterium to gain a foothold within the brain, while over-exuberant or protracted inflammation might cause substantial collateral damage to sensitive and non-renewable cells such as neurons. In this opinion piece, we compare host immunity against one prototype virus and one prototype bacterium known to cause disease either outside or within the CNS. Allowing for some variability in disease pathogenesis, and leaving aside complex issues related to chronic intrauterine or neonatal infections, we argue that antimicrobial host responses in both CNS and non-CNS tissue compartments of adult hosts who acquire these infections exhibit many more similarities than differences. In this setting, the concept of CNS immune privilege seems antiquated.

## Immune Surveillance of the Normal CNS and Mobilization of Host Responses during Acute CNS Infection

It is now accepted that there is a need for constant immune surveillance of the adult CNS as part of normal host defense, acknowledging that simultaneous mechanisms must keep local CNS inflammation strongly in check (1). Indeed, blockade of normal lymphocyte homing through the CNS can occasionally trigger local virus recrudescence (3), and low numbers of lymphocytes and antigen-presenting dendritic cells (DC) are found in perivascular spaces of the normal brain (4). For infectious particles that unexpectedly gain access to the CNS, some pathogen clearance occurs via bulk flow along paravenous routes by means of a process that depends on astrocytic water channels (4). This so-called “glymphatic system” of the brain ultimately carries antigenic material toward a specific group of large-caliber veins that drain to deep cervical lymph nodes (CLN) (5, 6). The CLN are increasingly appreciated as an important site where antigen-specific immune responses bound for the CNS are generated (7). Blood-borne pathogens, on the other hand, are mostly carried to the spleen where adaptive immunity occurs. Immune cells then migrate back to the CNS under the influence of chemokine gradients induced by infection, and bind and traverse the BBB via the actions of specific adhesion receptors and degradative enzymes.

## Infections in the Periphery or the CNS Caused by the Same Pathogen – How Much do Host Responses Differ?

*Streptococcus pneumoniae* is an important pathogen because it is the main cause of both community-acquired pneumonia and meningitis induced by a bacterial pathogen in otherwise healthy older children and adults. Much has also been learned about host immune responses elicited by pneumococcal pneumonia or meningitis using mouse models (8, 9). These infections develop in both mice and humans following pathogen inhalation and subsequent local tissue invasion (Table 1). Tissue-resident innate immune pathways are activated and host immunity is mobilized within hours. Outcome is determined over days to a few weeks. Morbidity and mortality, even in previously healthy hosts, is substantial, as many of the same mediators that have antibacterial activities also cause direct cellular damage (proteins, membrane lipids, DNA). Current polyvalent vaccines are effective in preventing both forms of invasive disease.

**Table 1:** **Pathogenesis and host responses elicited during a prototype bacterial (*Streptococcus pneumoniae*) or viral (lymphocytic choriomeningitis virus) infection of adult hosts when localized in either the periphery or the CNS [data adapted from Ref. (10–15)]^a^**.

	*Streptococcus pneumoniae*		Lymphocytic choriomeningitis virus	
	Pneumonia	Meningitis	Visceral infection (liver, spleen)	Meningitis
Natural routes of infection (humans)	Inhalation Local spread from nasopharyngeal colonization	Inhalation Local spread from an infected sinus or inner ear	Inhalation	Inhalation
			Direct contact with infected rodents	Direct contact with infected rodents
			Direct inoculation via infected solid organ transplant	Direct inoculation via infected solid organ transplant
Experimental routes of infection (mice)	Intranasal	Intranasal	Intravenous	Intracranial
		Intracisternal	Intraperitoneal	
Innate immune receptors activated	TLR2, TLR4, TLR9, NOD2, NRLP3	Unknown	TLR2, PKR, RLR, TLR7, MDA5	TLR2, CXCR3
Early innate immune mediators induced	IL-1β, TNF-α, IL-6	IL-1β, TNF-α, IL-6	IFN-α/β, TNF-α, IL-6, IL-10, CCL2, CCL5, CXCL10	IFN-α/β, CCL2, CCL3, CCL5, CXCL10
Site of main adaptive immune priming	Hilar/mediastinal lymph nodes	Cervical lymph nodes	Spleen	Spleen
	Spleen	Spleen	Mesenteric lymph nodes	Cervical lymph nodes
Principal effector cells activated and mobilized	Neutrophils	Neutrophils	CD8+ CTL	CD8+ CTL Monocytes
	Monocytes	Monocytes	NK cells	
	Dendritic cells	Dendritic cells	Dendritic cells	
	Lymphocytes	Lymphocytes		
Time to mobilize immune cells to target tissue	Hours	Hours	Hours–days	Hours–days
Soluble immune mediators involved in pathogen containment and/or clearance	IL-1β, TNF-α, NO, complement C1, IL-10	TNF-α, ROS, NO	IFN-α/β	IFN-α/β, CXCL10, IFN-γ
Mechanisms of pathogen clearance	Phagocytosis	Phagocytosis	Virus-specific CTL	Virus-specific CTL
	Neutrophil oxidative burst	Neutrophil oxidative burst	
	Complement activation	Complement activation	
Other relevant immune features	Disease severity and complications higher in asplenic individuals (humans)	Intracranial complications more common in asplenic individuals (humans)	Vicerotropic viral strains may cause chronic infection and immunosuppression via CTL exhaustion (mice)	No evidence of chronic CNS infection (humans)
	IκB and IL-10 polymorphisms raise susceptibility (humans)	
Potential for target tissue immunopathology (humans)	Moderate (10% overall mortality)	High (75% develop intracranial complications, 25% mortality)	Low (healthy adults)	Low (healthy adults)
			High (immunocompromised organ transplant recipients)	High (immunocompromised organ transplant recipients)
Potential for target tissue immunopathology (Mice)	High (most models cause lethal disease with extensive lung damage)	High	Moderate (adult mice)	High (adult mice infected with naturally occurring Armstrong strain)
Effectors of target tissue immunopathology	Lipocalin-2, NO, malondialdehyde, IL-1β, TNF-α	IFN-γ, TNF-α, glutamate, NO, ROS, caspase-9/3, myeloperoxidase	Virus-specific CTL, perforin	Virus-specific CTL, perforin
Role of immunotherapy in improved disease outcome	No proven role to date (humans)IVIG, MALP-2, and pneumococcal P4 peptide all improve survival (mice)	Corticosteroids of limited benefit to prevent hearing loss (humans)	No proven role to date (humans)	No proven role to date (humans)Virus-specific CTL plus virus-specific CD4+ T cells can clear persistent infection following adoptive transfer (mice)
		Inhibitors of caspases, ROS, IDO, kynurenine pathway improve cognitive outcomes (mice)		

*^a^Includes studies where peripheral and CNS responses were not directly compared, and therefore differences may reflect how the individual studies were conducted and what parameters were examined*.

*CCL, C–C motif ligand; CTL, cytotoxic T lymphocyte; CXCL, C–X–C motif ligand; CXCR, C–X–C motif receptor; IDO, indoleamine 2,3-dioxygenase; IFN, interferon; IL, interleukin; IVIG, intravenous immune globulin; MALP, macrophage-activating lipopeptide; MDA, melanoma differentiation-associated protein; NO, nitric oxide; NOD, nucleotide-binding oligomerization domain-containing protein; NRLP, NOD-like receptor family, pyrin domain-containing; PKR, protein kinase R; RLR, RIG-I-like receptor; ROS, reactive oxygen species; TLR, Toll-like receptor; TNF, tumor necrosis factor*.

Lymphocytic choriomeningitis virus (LCMV) is an arenavirus to which both mice and humans are susceptible and that causes varying combinations of visceral (hepatitis, pancreatitis, myocarditis) and/or CNS (meningitis, encephalitis) involvement in adult hosts. Murine LCMV infection has served as a prototype experimental system to study immunity to viruses for many years (10). This pathogen gets inhaled or directly inoculated into susceptible murine and human recipients (Table 1). Both innate and adaptive immune pathways are mobilized and disease can last several weeks. Healthy adults generally recover reasonably well, although the disease is more fulminant in immunocompromised hosts. In mice, the same virus-specific cytotoxic T cell (CTL) response that clears virus from infected tissues also causes tissue immunopathology. For acute CNS infection (choriomeningitis) in the setting of impaired CTL activity, survival is the trade-off for poor viral clearance.

The host responses provoked by these two naturally occurring pathogens are used here to compare how the immune system recognizes and responds to the same challenge in the periphery and the CNS (Table 1). The availability of mouse models for both pathogens that can be manipulated to cause infection either outside or within the CNS allows for the identification of immune determinants that directly influence disease outcome. It seems notable that many more similarities than differences in host immunity are seen, and that there is little evidence suggesting immune recognition is delayed or immune responses dampened for those infections localized primarily to the CNS compartment. Outcome differences for infections occurring at the two sites are likely driven by mechanisms independent of the host response.

## Conclusion

Both the afferent and efferent arms of the immune system are efficiently activated in the context of either pneumococcal or LCMV infection, and the kinetics, magnitude, and composition of immune responses elicited in adult hosts appear remarkably similar when either a visceral organ or the CNS is the main target of disease (Table 1). Acute CNS infections generally result in less favorable outcomes than those localized to the periphery, but this is more likely explained by the exquisite sensitivity of the brain to cellular damage rather than any delayed immune recognition behind the BBB. This comparison leads us to suggest that the concept of CNS immune privilege in the adult host seems somewhat obsolete in the setting of acute CNS infection.

## Conflict of Interest Statement

The authors declare that the research was conducted in the absence of any commercial or financial relationships that could be construed as a potential conflict of interest.
